# Jjj1 Is a Negative Regulator of Pdr1-Mediated Fluconazole Resistance in *Candida glabrata*

**DOI:** 10.1128/mSphere.00466-17

**Published:** 2018-02-21

**Authors:** Sarah G. Whaley, Kelly E. Caudle, Lucia Simonicova, Qing Zhang, W. Scott Moye-Rowley, P. David Rogers

**Affiliations:** a Department of Clinical Pharmacy, College of Pharmacy, University of Tennessee Health Science Center, Memphis, Tennessee, USA; b Department of Molecular Physiology and Biophysics, Carver College of Medicine, University of Iowa, Iowa City, Iowa, USA; Carnegie Mellon University

**Keywords:** *Candida glabrata*, antifungal resistance, fluconazole

## Abstract

The high prevalence of fluconazole resistance among clinical isolates of *Candida glabrata* has greatly hampered the utility of fluconazole for the treatment of invasive candidiasis. Fluconazole resistance in this yeast is almost exclusively due to activating mutations in the transcription factor Pdr1, which result in upregulation of the ABC transporter genes *CDR1*, *PDH1*, and *SNQ2* and therefore increased fluconazole efflux. However, the regulation of Pdr1 is poorly understood. In order to identify genes that interact with the Pdr1 transcriptional pathway and influence the susceptibility of *C. glabrata* to fluconazole, we screened a collection of deletion mutants for those exhibiting increased resistance to fluconazole. Deletion of the gene coding for a protein homologous to the *Saccharomyces cerevisiae* J protein Jjj1 resulted in decreased fluconazole susceptibility. We used the *SAT1* flipper method to generate independent deletion mutants for *JJJ1* in an SDD clinical isolate. Expression of both *CDR1* and *PDR1* was increased in the absence of *JJJ1*. In the absence of *CDR1* or *PDR1*, deletion of *JJJ1* has only a modest effect on fluconazole susceptibility. Transcriptional profiling using transcriptome sequencing (RNA-seq) revealed upregulation of genes of the Pdr1 regulon in the absence of *JJJ1*. Jjj1 appears to be a negative regulator of fluconazole resistance in *C. glabrata* and acts primarily through upregulation of the ABC transporter gene *CDR1* via activation of the Pdr1 transcriptional pathway.

**IMPORTANCE** *Candida glabrata* is the second most common species of *Candida* recovered from patients with invasive candidiasis. The increasing number of infections due to *C. glabrata*, combined with its high rates of resistance to the commonly used, well-tolerated azole class of antifungal agents, has limited the use of this antifungal class. This has led to the preferential use of echinocandins as empirical treatment for serious *Candida* infections. The primary mechanism of resistance found in clinical isolates is the presence of an activating mutation in the gene encoding the transcription factor Pdr1 that results in upregulation of one or more of the efflux pumps Cdr1, Pdh1, and Snq2. By developing a better understanding of this mechanism of resistance to the azoles, it will be possible to develop strategies for reclaiming the utility of the azole antifungals against this important fungal pathogen.

## Abstract (precis)

*Candida glabrata* is the second most common species of *Candida* recovered from patients with invasive candidiasis. The increasing number of infections due to *C. glabrata*, combined with its high rates of resistance to the commonly used, well-tolerated azole class of antifungal agents, has limited the use of this antifungal class. This has led to the preferential use of echinocandins as empirical treatment for serious *Candida* infections. The primary mechanism of resistance found in clinical isolates is the presence of an activating mutation in the gene encoding the transcription factor Pdr1 that results in upregulation of one or more of the efflux pumps Cdr1, Pdh1, and Snq2. By developing a better understanding of this mechanism of resistance to the azoles, it will be possible to develop strategies for reclaiming the utility of the azole antifungals against this important fungal pathogen.

## INTRODUCTION

*Candida glabrata* is the second most common cause of *Candida* infection (1, 2). Fluconazole has long been among frontline therapies for the treatment of invasive candidiasis. However, *C. glabrata* exhibits intrinsic reduced susceptibility to fluconazole and often develops high-level resistance during fluconazole therapy (3, 4). As such, the most recent clinical guidelines for treatment of candidiasis now recommend empirical therapy with an echinocandin rather than fluconazole in large part due to the problem of fluconazole resistance in this *Candida* species (5).

In *C. glabrata*, resistance to fluconazole is almost exclusively due to activating mutations in the gene encoding the zinc cluster transcription factor Pdr1. Pdr1 is the homolog of Pdr1 and Pdr3 in *Saccharomyces cerevisiae*, which regulates genes involved in the pleiotropic drug resistance phenotype. In *C. glabrata*, Pdr1 activates the expression of the genes encoding the ATP binding cassette (ABC) transporters Cdr1, Pdh1, and Snq2. It has been proposed that fluconazole can activate Pdr1 by binding directly to its xenobiotic binding domain (6). Moreover, Pdr1 can be activated by mitochondrial loss as is observed in “petite” mutants (7). While activation of Pdr1 in the presence of xenobiotics is dependent on binding of the activation domain of Pdr1 to the kinase-inducible domain interacting (KIX) domain of the mediator complex component Gal11a, our understanding of how Pdr1 is regulated in *C. glabrata* is incomplete (6).

In an effort to better understand this process, we screened a collection of 217 single gene deletion strains of *C. glabrata* for increased resistance to fluconazole (8). Deletion of the putative J protein CAGL0J07370g resulted in fluconazole resistance. This gene shares greatest homology with the *S. cerevisiae* gene *JJJ1*. We show here that disruption of *JJJ1* in a wild-type *C. glabrata* clinical isolate results in increased resistance to fluconazole through a Pdr1-dependent increased expression of the ABC transporter gene *CDR1*.

## RESULTS

### Deletion of *JJJ1* results in fluconazole resistance in a laboratory strain of *C. glabrata*.

We screened 217 strains from a previously published collection of mutants deleted for genes encoding putative transcription factors and DNA binding proteins for increased resistance to fluconazole (8). Three strains exhibited fluconazole MICs that were greater than 1 dilution higher than that of the parent strain (Table 1). Two mutants, those deleted for CAGL0K05797g (*EMI1*) and CAGL0C00297g (*ScSET2*), exhibited a fourfold increase in fluconazole MIC. One strain, deleted for CAGL0J07370g, exhibited a 16-fold increase in fluconazole MIC. CAGL0J07370g has sequence homology to the gene encoding the type III J protein Jjj1 in *S. cerevisiae*.

**TABLE 1:** Fluconazole MICs for single gene deletion mutant strains

*C. glabrata* strain or gene designation	*C. glabrata* gene name	*S. cerevisiae* orthologous gene	MIC_50_ (mg/liter)
Parent			4
CAGL0K05797g	*EMI1*	*EMI1*	16
CAGL0C00297g		*SET2*	16
CAGL0J07370g	*JJJ1*	*JJJ1*	64

### Deletion of *JJJ1* in a susceptible-dose dependent clinical isolate of *C. glabrata* confers resistance to fluconazole.

**FIG 1:**
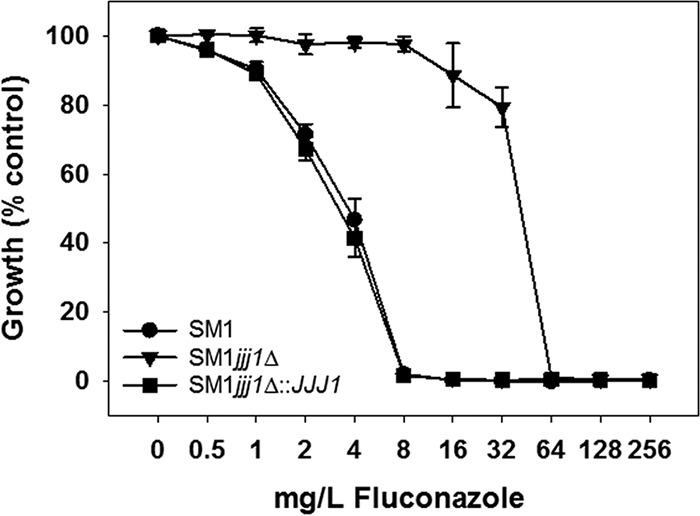
Deletion of *JJJ1* in the susceptible-dose dependent clinical isolate SM1 results in decreased fluconazole susceptibility. Reintegration of *JJJ1* into its original locus restored the susceptible-dose dependent phenotype. Strains were grown in 96-well plates according to standard CLSI methods with minor modifications, and optical density at 600 nm was measured after 48 h.

To confirm the phenotype of the *JJJ1* mutant strain, we generated an additional *JJJ1* deletion strain in clinical isolate SM1 using the *SAT1* flipper method (9). Isolate SM1 was originally recovered from an antifungal-naive patient undergoing cancer chemotherapy (10). Deletion of *JJJ1* in this susceptible-dose dependent (SDD) clinical isolate resulted in a 16-fold increase in MIC to fluconazole—from 4 mg/liter to 64 mg/liter (Table 2 and Fig. 1). This increase was identical to that observed in the *JJJ1* mutant strain from the deletion collection. We then constructed a complemented derivative strain using the *SAT1* flipper method. The MIC of fluconazole upon reintroduction of *JJJ1* was restored to that of the parent, clinical isolate SM1.

**TABLE 2:** Fluconazole MICs for indicated strains

Strain	MIC_50_ (mg/liter)
SM1	4
SM1*jjj1*Δ	64
SM1*jjj1*Δ::*JJJ1*	4
SM1*cdr1*Δ	1
SM1*pdr1*Δ	1
SM1*jjj1*Δ*cdr1*Δ	2
SM1*jjj1*Δ*pdr1*Δ	2

### Jjj1-mediated fluconazole resistance is dependent upon Cdr1.

**FIG 2:**
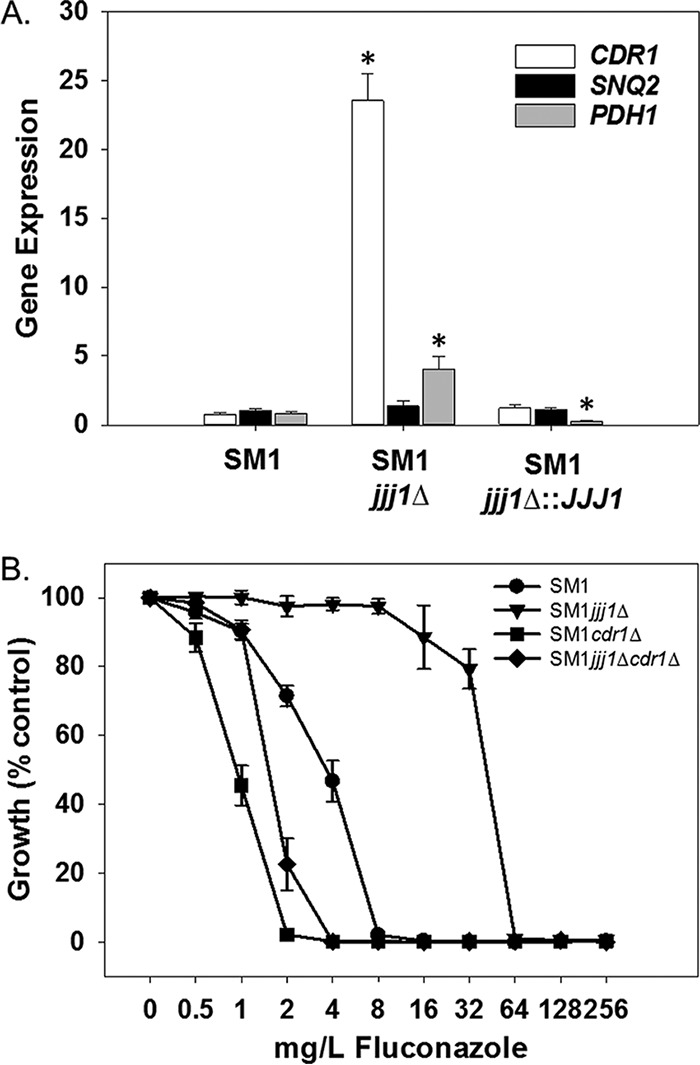
*JJJ1* influences fluconazole susceptibility in a *CDR1*-dependent manner. (A) The effects of *JJJ1* deletion on expression of the genes encoding the ABC transporters Cdr1, Snq2, and Pdh1 was measured by qRT-PCR. Expression was normalized to 18S rRNA expression in the parent isolate SM1. Changes were compared using a Student’s *t* test. Gene expression values marked with an asterisk are statistically significant (*P* < 0.05). (B) Strains were grown in 96-well plates according to standard CLSI methods with minor modifications, and optical density at 600 nm was measured after 48 h.

In clinical isolates of *C. glabrata*, fluconazole resistance is mediated by the ABC transporters Cdr1, Pdh1, and Snq2 (11–14). To determine whether increased fluconazole resistance observed when *JJJ1* is deleted is also mediated by these transporters, we first measured the expression levels of their respective genes using quantitative real-time PCR (qRT-PCR). Deletion of *JJJ1* in clinical isolate SM1 resulted in *CDR1* expression more than 20-fold and *PDH1* expression more than 4-fold that observed in the parent strain (Fig. 2A). There was no significant change in the expression of *SNQ2*.

We then deleted *CDR1* in the *JJJ1* deletion mutant. Deletion of *CDR1* resulted in greater susceptibility to fluconazole than the wild-type parent isolate SM1 (Fig. 2B). However, this mutant was not as susceptible as a *CDR1* deletion mutant in the SM1 background. These observations suggest that the increased fluconazole resistance observed upon deletion of *JJJ1* is due in large part to *CDR1*, but that other determinants, possibly *PDH1*, contribute modestly to this phenotype as well.

### Jjj1-mediated fluconazole resistance and *CDR1* expression is dependent on Pdr1.

**FIG 3:**
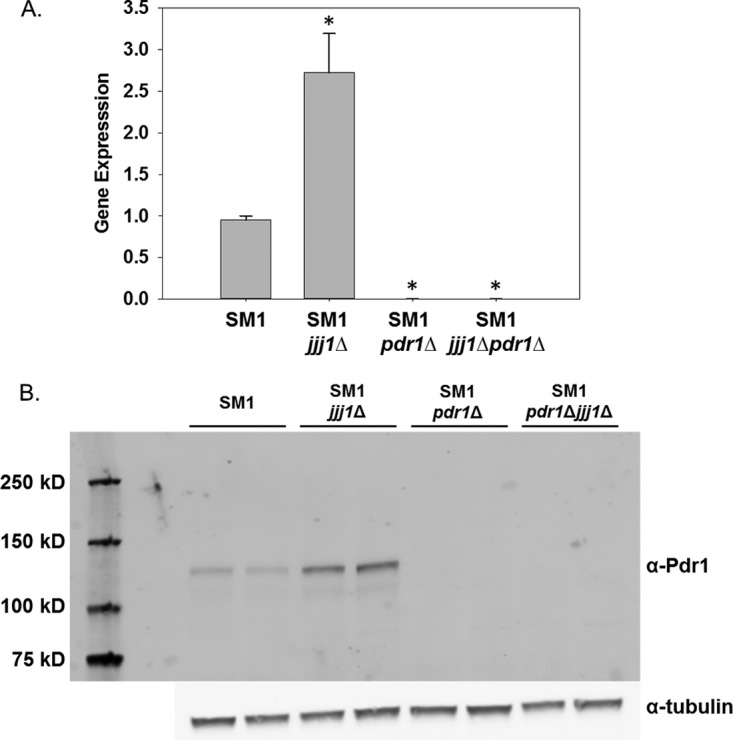
*JJJ1* deletion results in altered expression of *PDR1* at both transcript and protein levels. (A) The effects of *JJJ1* and *PDR1* deletion alone and in combination on expression of the gene encoding the transcription factor Pdr1 were measured by qRT-PCR. Expression was normalized to 18S rRNA expression in the parent isolate SM1. Changes were compared using a Student’s *t* test. Gene expression values marked with an asterisk are statistically significant (*P* < 0.05). (B) The effect of *JJJ1* and *PDR1* deletion alone and in combination on protein levels of Pdr1 was assessed by Western blot analysis. The positions of molecular mass markers (in kilodaltons) are shown to the left of the gel. α-Pdr1, anti-Pdr1 antibody.

*CDR1* is a direct target of the zinc cluster transcription factor Pdr1. The expression of *CDR1* in response to fluconazole requires activation of Pdr1 and overexpression of *CDR1* in fluconazole-resistant clinical isolates is due to activating mutations in *PDR1* (7, 11, 15). Of note, *PDR1* is autoregulated (7, 16), so we predicted that deletion of *JJJ1* would result in upregulation of *PDR1* gene expression and concomitant increased Pdr1 protein expression. *PDR1* expression increased 2.7-fold in the *JJJ1* knockout compared to that of the parent strain (Fig. 3A). As expected, there is no *PDR1* expression in the strain with *PDR1* deleted alone or in the strain with both *JJJ1* and *PDR1* deleted. Pdr1 protein levels followed the same pattern (Fig. 3B).

**FIG 4:**
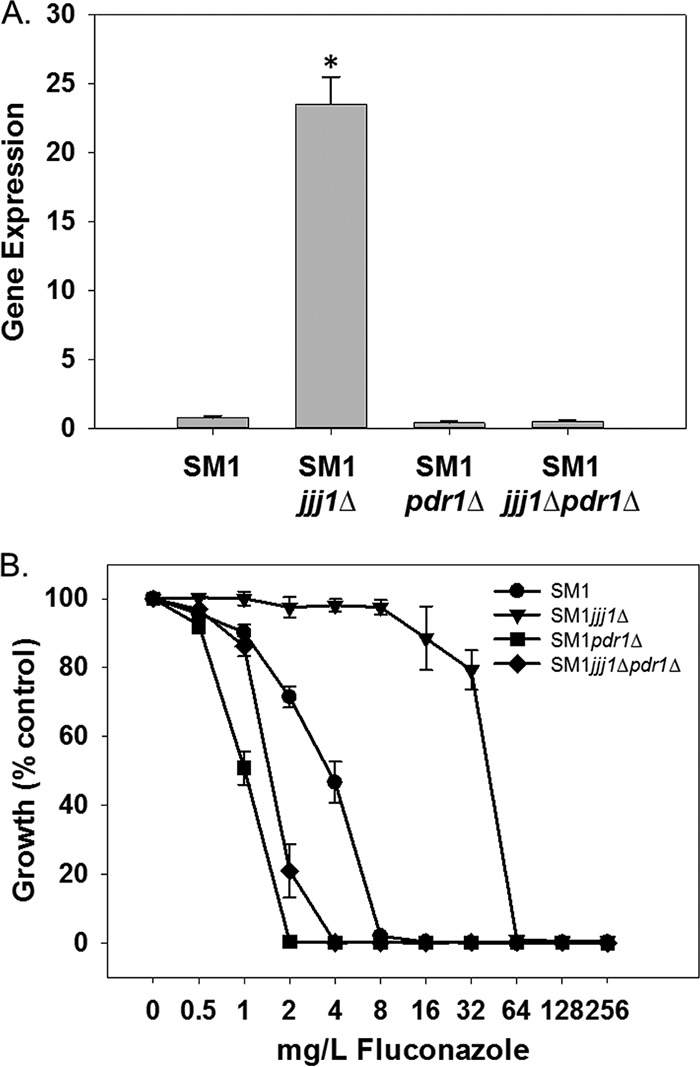
*JJJ1* influences fluconazole susceptibility in a *PDR1*-dependent manner. (A) The effects of *JJJ1* and *PDR1* deletion alone and in combination on expression of the genes encoding the ABC transporter Cdr1 were measured by qRT-PCR. Expression was normalized to 18S rRNA expression in the parent isolate SM1. Changes were compared using a Student’s *t* test. Gene expression values marked with an asterisk are statistically significant (*P* < 0.05). (B) Strains were grown in 96-well plates according to standard CLSI methods with minor modifications, and optical density at 600 nm was measured after 48 h.

To determine whether the effects on *CDR1* expression and fluconazole susceptibility observed upon deletion of *JJJ1* are dependent upon activation of Pdr1, we deleted *PDR1* in the *JJJ1* deletion mutant and measured expression of *CDR1* and susceptibility to fluconazole. Deletion of *PDR1* reduced expression of *CDR1* in the absence of *JJJ1* to levels observed in the wild-type parent strain (Fig. 4A). Deletion of *PDR1* in the *JJJ1* deletion mutant increased fluconazole susceptibility beyond what was observed in the wild-type parent strain (Fig. 4B), but not to the extent of that observed in the *PDR1* deletion mutant. This suggests that while most of the effect of deleting *JJJ1* on fluconazole susceptibility is dependent upon Pdr1, some of the effects of Pdr1 on fluconazole susceptibility are not affected by Jjj1.

### Deletion of *JJJ1* activates genes of the Pdr1 regulon.

In order to determine what genes in addition to *CDR1*, *PDH1*, and *PDR1* are differentially expressed when *JJJ1* is deleted, we used transcriptome sequencing (RNA-seq) to compare the transcriptional profiles of both the *JJJ1* deletion mutant and the *JJJ1*/*PDR1* deletion mutant to that of parent isolate SM1. In the *JJJ1* deletion mutant compared to the parent strain, 204 genes were upregulated and 224 genes were downregulated by 1.5-fold or greater (see Tables S1 and S2 in the supplemental material). Upregulation and downregulation of 119 and 149 of these upregulated and downregulated genes, respectively, required *PDR1* (Table S1 and S2, boldface genes). As expected, we observed *CDR1*, *PDH1*, and *PDR1* to be among those genes whose upregulation upon deletion of *JJJ1* required *PDR1*. Of the 25 targets of Pdr1 that have been previously confirmed by chromatin immunoprecipitation sequencing (ChIP-seq) (17), 7 were found to be upregulated when *JJJ1* was deleted. These targets were *CDR1*, *YBT1*, *YOR1*, *RSB1*, *RTA1*, *PDH1*, and *NCE103*. One of these targets, *NCE103*, remained upregulated in the absence of both *JJJ1* and *PDR1*. Of the 85 genes upregulated in the absence of both *JJJ1* and *PDR1*, 7 are involved in methionine biosynthesis (*MET6*, *MUP1*, *MET8*, *MET13*, *S*. *cerevisiae MET2* [*ScMET2*], *ScMXR1*, and *MET15*). Seventeen genes predicted to have a role in adhesion were observed to be upregulated in the absence of *JJJ1*, four of which required *PDR1* (Table S3).

10.1128/mSphere.00466-17.1 TABLE S1  Genes upregulated by at least 1.5-fold in SM1*jjj1*Δ compared to SM1. Fold change is defined as the ratio of gene expression in SM1*jjj1*Δ compared to SM1 in two independent RNA-seq experiments. Boldface rows indicate genes whose upregulation is dependent on *PDR1* expression. Download TABLE S1, XLSX file, 0.03 MB. Copyright © 2018 Whaley et al. 2018 Whaley et al. This content is distributed under the terms of the Creative Commons Attribution 4.0 International license.

10.1128/mSphere.00466-17.2 TABLE S2  Genes downregulated by at least 1.5-fold in SM1*jjj1*Δ compared to SM1. Fold change is defined as the ratio of gene expression in SM1*jjj1*Δ compared to SM1 in two independent RNA-seq experiments. Boldface rows indicate genes whose downregulation is dependent on *PDR1* expression. Download TABLE S2, XLSX file, 0.03 MB. Copyright © 2018 Whaley et al. 2018 Whaley et al. This content is distributed under the terms of the Creative Commons Attribution 4.0 International license.

10.1128/mSphere.00466-17.3 TABLE S3  Adhesion-related genes upregulated by at least 1.5-fold in SM1*jjj1*Δ compared to SM1. Fold change is defined as the ratio of gene expression in SM1*jjj1*Δ compared to SM1 in two independent RNA-seq experiments. Boldface rows indicate genes whose upregulation is dependent on *PDR1* expression. Download TABLE S3, XLSX file, 0.01 MB. Copyright © 2018 Whaley et al. 2018 Whaley et al. This content is distributed under the terms of the Creative Commons Attribution 4.0 International license.

## DISCUSSION

Unlike other species of *Candida*, azole resistance in clinical isolates of *C. glabrata* is almost exclusively due to activating mutations in the gene encoding the transcription factor Pdr1 that lead to increased expression of the genes encoding the ATP binding cassette (ABC) transporters *CDR1*, *PDH1*, and *SNQ2*. Single amino acid substitutions in Pdr1 can result in its activation, and the effects on expression of downstream target genes vary depending on the mutation (18–20). This lends itself to a hypothesis that Pdr1 is negatively regulated and that single amino acid changes interfere with the negative regulation, resulting in altered gene expression. The varied patterns of gene expression seen with the different activating Pdr1 mutations would indicate that more than one negative regulatory mechanism may exist.

Regulation of Pdr1 in *C. glabrata* is not fully understood, but recent work in this area provides some insight. The transcription factor Stb5 has been shown to be a negative regulator of Pdr1. Deletion of *STB5* in a wild-type background resulted in minimal decreased susceptibility; however, in a *pdr1*Δ mutant strain, deletion of *STB5* resulted in marked decreased susceptibility to the azoles. Overexpression of *STB5* increased azole susceptibility. In addition, the expression profile of the *STB5* deletion strain overlaps with that of a mutant strain overexpressing *PDR1* (21). In the closely related nonpathogenic yeast *Saccharomyces cerevisiae*, Stb5 forms a heterodimer with the transcription factor Pdr1 and binds the promoter of the ABC transporter *PDR5* directly (22). However, susceptibility to ketoconazole was not shown to be affected in an *S. cerevisiae STB5* deletion strain (23).

Pdr1 is also regulated at the level of transcription through the mediator complex. Deletion of *GAL11A*, which codes for a member of the mediator complex, results in decreased expression of the ABC transporter *PDH1* and increased azole sensitivity. A direct interaction between the Gal11a KIX domain and Pdr1 has been demonstrated (6). Pdr1 was shown to act as a xenobiotic receptor and bind ketoconazole directly. Gal11a is important for drug-induced Pdr1 activation; however, it is dispensable for Pdr1 activation in petite mutants (16). Pdr1 is also autoregulated through binding of the pleiotropic drug response element (PDRE) located in its promoter region (16, 17).

In *S. cerevisiae*, two zinc finger transcription factors, Pdr1 and Pdr3, are responsible for regulation of the pleiotropic drug response. Understanding how this regulation occurs is informative for forming a model for regulation in *C. glabrata*, which has not been studied as thoroughly thus far. Pdr1 and Pdr3 regulation occurs through similar yet distinct pathways.

Pdr1 function in *S. cerevisiae* is regulated by the Hsp70/Hsp40 cochaperone pair Ssz1/Zuo1. Ssz1 and Zuo1 are part of a ribosome-associated complex that is involved in folding of newly synthesized proteins (24); however, this activity is distinct from that involved in regulation of Pdr1 and the multidrug resistance phenotype (25, 26). Ssz1 and Zuo1 are both able to activate Pdr1 independently of one another, indicating that they are not acting as chaperones in this case (25). A region at the C terminus of Zuo1 has been shown to bind directly to Pdr1, similar to xenobiotic direct binding of Pdr1 (26). Overexpression of Ssz1 leads to an increase in Pdr1 target genes and increases tolerance to cycloheximide and oligomycin, indicating that it acts as a positive regulator (27).

Pdr3 in *S. cerevisiae* is also regulated by an Hsp70, but in this case it appears to be negative regulation. Overexpression of the Hsp70 gene *SSA1* leads to increased sensitivity to cycloheximide and decreased Pdr1 target gene expression (28). Previous work had shown that Pdr3 is activated in mitochondrion-deficient mutants (29). There is less Ssa1 associated with Pdr3 in these mutants, indicating that this regulatory pathway is involved in the altered drug susceptibility associated with mitochondrial insufficiency. Deletion of the nucleotide exchange factor Fes1, which is thought to inhibit Ssa1 activity also increased sensitivity to cycloheximide, but no Hsp40 working in conjunction with the Hsp70 Ssa1 has been described (28).

The *C. glabrata* open reading frame (ORF) CAGL0J07370g identified in our screen to affect fluconazole susceptibility has the characteristic J domain present in members of the Hsp40 class of proteins. The primary role for Hsp40 proteins attributed to the J domain is stimulation of ATP hydrolysis through interaction of Hsp70 ATPase domains (30, 31). The closest homolog to CAGL0J07370g is *ScJJJ1* from *S. cerevisiae*, which shares 66. 2% amino acid similarity and 51.2% identity as calculated using EMBOSS Needle (32). *ScJJJ1* and *ScZUO1* have the J domain in common, as well as another region thought to bind the ribosome, which is unique to these two genes among all Hsp40s in *S. cerevisiae* (33, 34). Importantly, deletion of *ScJJJ1* results in increased sensitivity to the azoles, which is the opposite effect from that observed upon deletion of CAGL0J07370g in *C. glabrata* (35).

The experiments described here demonstrate a role for *JJJ1* in fluconazole susceptibility in *C. glabrata*. Deletion of *JJJ1* in a susceptible-dose dependent isolate results in fluconazole resistance. This altered susceptibility is primarily a result of *PDR1*-dependent activation of *CDR1*. On the basis of what is known in the closely related species *S. cerevisiae* as well as what is known in *C. glabrata*, we propose a model for the role of the Hsp40 Jjj1 in Pdr1 regulation. Posttranscriptionally, Pdr1 is negatively regulated by Jjj1, and this may involve an Hsp70, a nucleotide exchange factor, or both. When the Jjj1/Pdr1 interaction is disrupted, Pdr1 is activated and able to upregulate a distinct set of target genes.

Our transcriptional profiling data support this proposed mechanism. Chromatin immunoprecipitation combined with sequencing has been used to determine the direct binding targets of Pdr1 (17). Eight genes whose altered expression in the *JJJ1* mutant is dependent on *PDR1* are direct Pdr1 targets—six exhibited upregulation (*CDR1*, *YBT1*, *YOR1*, *RSB1*, *RTA1*, and *PDH1*) and two were downregulated (*ATF2* and *ScBAG7*). Only one known direct Pdr1 target, *NCE103*, showed altered expression that was independent of *PDR1* expression. Additional indirect Pdr1 targets identified by previously published microarray data are also among the genes upregulated in the *JJJ1* deletion strain in a *PDR1*-dependent manner (*ScGPP1*, *ScCIS1*, *ScLAC1*, *ScMCP1*, *ScGUT2*, *ScPBI2*, *ScGSF2*, *GLK1*, and *ILV5*) (18, 20, 36, 37).

While the Pdr1 pathway appears to be primarily responsible for the altered gene expression in the *JJJ1* deletion strain, there is a consistent 1 dilution change in MIC when *JJJ1* is deleted in strains lacking *CDR1* or *PDR1*. This finding allows for the possibility that there may be Pdr1-independent effects as well.

Among the genes upregulated in the *JJJ1* deletion strain that were independent of *PDR1* expression were many adhesion-related genes. Five members of the *EPA* family were in this group. Three of these genes, *EPA1*, *EPA2*, and *EPA3*, are part of a cluster of genes whose transcription is controlled by subtelomeric silencing (38, 39). Adhesins are known to be upregulated when nicotinic acid is limited (40); however, that does not appear to be happening in this experiment. None of the genes known to exhibit increased expression under nicotinic acid-limited conditions, for example *TNA1*, *TNR1*, and *TNR2*, have increased expression. Of particular interest among the adhesin genes found to be upregulated, *EPA1* has a role in increased adhesion to epithelial cells in strains of *C. glabrata* with activating mutations in *PDR1* (41). *EPA1* has the putative PDRE site in its promoter (41), but Pdr1 does not bind tightly (17). *EPA1* and many other adhesins were still upregulated in the mutant strain lacking *JJJ1* and *PDR1*. In addition to the *EPA* genes were *PWP1* and *PWP3*, which belong to adhesin cluster II. In total, there are 17 genes predicted to have a role in adhesion among the upregulated genes in the *JJJ1* deletion strain; for 13 of these genes, the increased expression is independent of Pdr1.

The experiments described here provide further insight into regulation of Pdr1 in the important fungal pathogen *C. glabrata*. Our data suggest that the J protein Jjj1 acts as a negative regulator of fluconazole resistance primarily through transcription factor Pdr1 and its target ABC transporter Cdr1.

## MATERIALS AND METHODS

### Strains and growth conditions.

Strains used in this study are listed in Table 3. The parent clinical isolate and the *PDR1* deletion strain have been described previously (10, 18). All strains were stored as frozen stocks at −80°C in 40% glycerol. Strains were routinely grown in yeast extract peptone dextrose (YPD) (1% yeast extract, 2% peptone, and 2% dextrose) broth at 30°C in a shaking incubator except as indicated for specific experimental conditions.

**TABLE 3:** Strains used in this study

Strain	Parent	Description or relevant genotype	Reference
SM1		Azole-SDD clinical isolate	10
SM1*jjj1*Δ	SM1	*jjj1*Δ::*FRT*	This study
SM1*jjj1*Δ::*JJJ1*	SM1*jjj1*Δ	*jjj1*Δ::*JJJ1-FRT*	This study
SM1*cdr1*Δ	SM1	*cdr1*Δ::*FRT*	This study
SM1*pdr1*Δ	SM1	*pdr1*Δ::*FRT*	18
SM1*jjj1*Δ*cdr1*Δ	SM1*jjj1*Δ	*jjj1*Δ::*FRT/cdr1*Δ::*FRT*	This study
SM1*jjj1*Δ*pdr1*Δ	SM1*pdr1*Δ	*jjj1*Δ::*FRT/pdr1*Δ::*FRT*	This study

*Escherichia coli* TOP10 One Shot chemically competent cells (Invitrogen, Carlsbad, CA) were used as the host for plasmid construction and propagation. These strains were grown at 37°C in LB broth or on LB plates supplemented with 100 μg/ml ampicillin (Sigma, St. Louis, MO) or 50 μg/ml kanamycin (Fisher BioReagents, Fair Lawn, NJ).

### Deletion library screen.

We obtained 217 *C. glabrata* single gene deletion strains from a previously published collection (8). These genes included genes encoding putative transcription factors and DNA binding proteins. The mutant strains were generated in a histidine auxotrophic mutant of *C. glabrata* strain ATCC 2001 (CBS138). Fluconazole MICs were determined for each deletion strain according to the Clinical and Laboratory Standards Institute (CLSI) reference method with minor modifications as described below (42, 43). Strains were tested at concentrations ranging from 64 mg/liter to 0.125 mg/liter fluconazole in duplicate.

### Plasmid construction.

For deletion of *JJJ1* and *CDR1*, we modified plasmid pSFS2 (9). Upstream homology regions approximately 800 to 1,000 bp long were amplified using primer pair CgJJJ1-A/CgJJJ1-B or CgCDR1-A/CgCDR1-B and digested with ApaI and XhoI for insertion into their respective plasmids. Downstream homology regions approximately 900 to 1,000 bp long were amplified using primer pair CgJJJ1-C/CgJJJ1-D or CgCDR1-C/CgCDR1-D and digested with SacI and SacII for insertion into their respective plasmids. The disruption cassettes consisting of the *SAT1* flipper cassette and upstream and downstream flanking sequences of either *JJJ1* or *CDR1* were excised from the final plasmid pCgJJJ1 or pCgCDR1 and gel purified. Primers used to construct the cassettes are listed in Table 4.

**TABLE 4:** Primers used in this study

Application and primer^a^	Primer sequence^b^
Cassettes for constructing mutants	
CgJJJ1-A	5′-AATTACAAAGGGCCCTATTTGAGTTACAGC-3′
CgJJJ1-B	5′-ATTATCTGGATTCTCGAGAGGATGATAC-3′
CgJJJ1-C	5′-AAGTAGGAATCCGCGGTTTAGTCATATACA-3′
CgJJJ1-D	5′-TATTTATGCTACGAGCTCTATTGACGTTAT-3′
CgJJJ1-E	5′-GTTTCCAAGCAACTCGAGATGATTAGT-3′
CgCDR1-A	5′-CATAGATCAGGGCCCATTACATTAGCACAG-3′
CgCDR1-B	5′-CTCAGTGTTGCTCGAGATAGGGTTGATAC-3′
CgCDR1-C	5′-GTTCTGTTAGTTCCGCGGACTCTCGTAGAT-3′
CgCDR1-D	5′-GTGAATACAAACAAGAGCTCCACAATAATA-3′

qRT-PCR	
18SF	5′-TCGGCACCTTACGAGAAATCA-3′
18SR	5′-CGACCATACTCCCCCCAGA-3′
PDR1F	5′-TTTGACTCTGTTATGAGCGATTACG-3′
PDR1R	5′-TTCGGATTTTTCTGTGACAATGG-3′
CDR1F	5′-CATACAAGAAACACCAAAGTCGGT-3′
CDR1R	5′-GAGACACGCTTACGTTCACCAC-3′
SNQ2F	5′-CGTCCTATGTCTTCCTTACACCATT-3′
SNQ2R	5′-TTTGAACCGCTTTTGTCTCTGA-3′
PDH1F	5′-ACGAGGAGGAAGACGACTACGA-3′
PDH1R	5′-CTTTACTGGAGAACTCATCGCTGGT-3′

a Primers are grouped by application. For the primers used for qRT-PCR, forward (F) and reverse (R) primers are indicated at the end of the primer name.

b Restriction enzyme cloning sites introduced to allow directional cloning into the *SAT1* flipper cassette are underlined.

For reintegration of *JJJ1* into *jjj1Δ* mutants, we modified plasmid pCgJJJ1. The open reading frame in addition to some upstream and downstream sequence was amplified using primer pair CgJJJ1-A/CgJJJ1-E and digested with ApaI and XhoI. This replaced the upstream homology region from plasmid pCgJJJ1, such that upon transformation and homologous recombination, *JJJ1* would be reintegrated into its original locus. The resulting plasmid is called pCgJJJ1pb.

### Strain construction.

*C. glabrata* cells were transformed by the lithium acetate method using approximately 1 μg of DNA. The ApaI/SacI fragments from pCgJJJ1, pCgCDR1, and pCgJJJ1pb were excised and gel purified prior to transformation. The transformed cells were allowed to recover 6 h in YPD at 30°C before being plated on YPD agar plates containing 200 μg/ml nourseothricin (Jena Biochemical, Germany) and incubated at 30°C. Positive transformants were selected within 24 h, and successful insertion of the disruption cassette at the target gene locus was confirmed by Southern hybridization using gene-specific probes. Subsequently, induction of the flipper recombinase gene in the disruption cassette was performed by overnight growth of the positive transformant clones in YPD at 30°C with shaking (under no selective pressure). Selection for excision of the *SAT1* flipper cassette was then performed by plating on YPD agar plates and incubating for up to 24 h at 30°C. Clones were selected and confirmed by Southern hybridization using gene-specific probes.

### Genomic DNA isolation and Southern analysis.

Genomic DNA from *C. glabrata* was isolated as described previously (44). For confirmation by Southern hybridization, approximately 10 μg of genomic DNA was digested with the appropriate restriction enzymes, separated on a 1% agarose gel containing ethidium bromide, transferred by vacuum blotting onto a nylon membrane and fixed by UV cross-linking. Hybridization was performed with the Amersham ECL direct nucleic acid labeling and detection system (GE Healthcare, Pittsburg, PA) per the manufacturer’s instructions.

### Susceptibility testing.

Susceptibility testing was performed by broth microdilution assay according to the CLSI guidelines outlined in approved standard M27-A3 with a few modifications (42, 43). Fluconazole (MP Biomedicals, Salon, OH) stock solution was prepared by reconstitution in water to 5 mg/ml. Cultures were diluted to 2. 5 × 10^3^ cells/ml in RPMI 1640 (Sigma, St. Louis, MO) containing 2% glucose and morpholinepropanesulfonic acid (MOPS) (pH 7.0). The plates were incubated at 35°C for 48 h. Absorbance at 600 nm was read with a BioTek Synergy 2 microplate reader (BioTek, Winooski, VT); background due to medium was subtracted from all readings. The MIC was defined as the lowest concentration inhibiting growth by at least 50% relative to the drug-free control after incubation with drug for 48 h.

### RNA isolation.

The RNA isolation procedure was the same for both quantitative real-time PCR (qRT-PCR) and transcriptome sequencing (RNA-seq) experiments. Log-phase cultures grown in YPD medium at 30°C were adjusted to an optical density at 600 nm (OD_600_) of 0.2, and the cultures were incubated for an additional 3 h to mid-log phase. RNA was extracted by the hot phenol method (45), as previously described (46). RNA was treated with RQ1 DNase (Promega, Madison, WI). The quantity and purity of RNA were determined by using a spectrophotometer (NanoDrop Technologies, Inc., Wilmington, DE) and verified using a Bioanalyzer 2100 (Agilent Technologies, Santa Clara, CA).

### Quantitative real-time PCR.

Quantitative RT-PCR was conducted as described previously (46). Single-strand cDNA was synthesized from 2 μg of total RNA using the Superscript first-strand synthesis system for RT-PCR (Invitrogen, Carlsbad, CA) according to the manufacturer’s instructions. Relative quantitative real-time PCRs were performed in triplicate using the 7000 Sequence Detection System (Applied Biosystems, Foster City, CA). Independent PCRs were performed using the primers listed in Table 4 for both the genes of interest and 18S rRNA using SYBR green PCR master mix (Applied Biosystems). Relative gene expression was calculated by the comparative (*C*_*T*_) method (ΔΔ*C*_*T*_ method). For expression of *PDR1*, *CDR1*, *SNQ2*, and *PDH1*, samples were normalized first to 18S rRNA expression and then to the parent strain SM1. Statistical analysis was performed using Microsoft Office Excel 2016. Relative changes were compared using a Student’s *t* test.

### Protein isolation and Western blot analysis.

Log-phase cells grown in YPD at 30°C were diluted to an OD_600_ of 0.2 and were grown in YPD for an additional 3 h. Three OD_600_ units of culture were harvested per sample, and two colonies of each strain were analyzed. Protein extracts were prepared as previously described (47). Protein pellets were resuspended in urea sample buffer (8 M urea, 1% 2-mercaptoethanol, 40 mM Tris-HCl [pH 8.0], 5% sodium dodecyl sulfate [SDS], bromophenol blue) and boiled at 90°C for 10 min. An aliquot from each sample was resolved on a precast ExpressPlus 4 to 15% gradient gel (GenScript) following the manufacturer’s SDS-polyacrylamide gel electrophoresis (PAGE) protocol. Proteins were electroblotted onto a nitrocellulose membrane, blocked with 5% nonfat dry milk, and then probed with anti-Pdr1 antibody (17) diluted 1:1,500. All membranes were probed for tubulin as the loading control with 12G10 anti-alpha-tubulin monoclonal antibody (Developmental Studies Hybridoma Bank at the University of Iowa) for 30 min at room temperature. The membrane was probed with secondary Li-Cor antibodies IRDye 680LT goat anti-rabbit diluted 1:20,000 and IRDye 800CW goat anti-mouse diluted 1:10,000. Western blot signal was detected using the Li-Cor Odyssey infrared imaging system, application software version 3. 0.

### RNA sequencing.

Bar-coded libraries were prepared using the Lexogen mRNA Sense kit for Ion Torrent according to the manufacturer’s standard protocol. Libraries were sequenced on the Ion Torrent Proton sequencer. Individual sample fragments were concatenated to form the whole-sample fastq file. Files were then run through FASTQC to check data quality. Any reads with a phred score of <20 were trimmed. After trimming, reads were aligned to the *C. glabrata* CBS138 reference transcriptome using RNA-Star long method. After alignment, transcriptome alignment counts were gathered. The read counts for each sample were normalized using transcripts per kilobase million (TPM) method.

### Data availability.

The RNA sequencing data were deposited in the Gene Expression Omnibus (GEO) database under accession number GSE104476.
